# Observing the climate impact of large wildfires on stratospheric temperature

**DOI:** 10.1038/s41598-021-02335-7

**Published:** 2021-11-26

**Authors:** Matthias Stocker, Florian Ladstädter, Andrea K. Steiner

**Affiliations:** 1 Wegener Center for Climate and Global Change (WEGC), University of Graz, 8010 Graz, Austria; 2 Institute for Geophysics, Astrophysics, and Meteorology/Institute of Physics, University of Graz, 8010 Graz, Austria

**Keywords:** Climate sciences, Atmospheric science, Natural hazards

## Abstract

Wildfires are expected to become more frequent and intense in the future. They not only pose a serious threat to humans and ecosystems, but also affect Earth’s atmosphere. Wildfire plumes can reach into the stratosphere, but little is known about their climate impact. Here, we reveal observational evidence that major wildfires can have a severe impact on the atmospheric temperature structure and short-term climate in the stratosphere. Using advanced satellite observation, we find substantial warming of up to 10 K of the lower stratosphere within the wildfire plumes during their early development. The short-term climate signal in the lower stratosphere lasts several months and amounts to 1 K for the Northern American wildfires in 2017, and up to striking 3.5 K for the Australian wildfires in 2020. This is stronger than any signal from recent volcanic eruptions. Such extreme events affect atmospheric composition and climate trends, underpinning their importance for future climate.

## Introduction

Wildfires influence the climate system by changing the surface albedo and releasing trace gases such as carbon dioxide as well as aerosols. The latter have a direct or indirect influence on the radiative fluxes in the atmosphere by changing cloud properties and atmospheric chemistry^1^. On a regional scale, aerosols can alter the radiative balance, thereby affecting surface air temperature, pressure, surface winds, and the stability of the planetary boundary layer^2,3^. The effects of wildfire aerosols on regional climate are complex and strongly depend on altitude, ranging from surface warming by aerosols near the ground to surface cooling by aerosols at high altitudes^4^. In this context wildfire emissions can be as important as those from industrial production^1,5^. Conversely, changes in the climate variability and climate warming can affect the occurrence and intensity of wildfires^1^.

One aspect that has received little attention so far is the potential of large wildfires to cause temperature changes in the stratosphere. There, the emitted aerosols are distributed over the whole hemisphere and linger for months to years, potentially affecting climate in the short term^6–8^.

Intense fires can trigger deep convection, depending on conditions such as the prevailing weather or the amount of heat released by the fire and during plume development^7^. In the presence of an additional moisture source in the mid-troposphere providing additional latent heat, intense pyrocumulonimbus (pyroCb) clouds can form. These pyroCb clouds form an extremely deep convective smoke column that can transport combustion products up into the stratosphere^9^.

While a large fraction of Earth’s land surface can be affected by wildfires, pyroCb plumes reaching the stratosphere develop most likely in temperate and boreal northern and southern hemispheric forests as they are found in Siberia, Northern America, and Australia^5^.

Intense wildfires have been observed to regionally alter stratospheric aerosol optical thickness and radiative forcing as well as stratospheric ozone concentrations in ways that were previously only known from moderate to large volcanic eruptions, with substantial effects on temperature in the lower stratosphere^8,10–13^. Compared to volcanic sulfate aerosols, however, wildfire smoke contains high amounts of organic carbon and black carbon, which strongly absorb at visible wavelengths and therefore have a strong heating potential^14,15^.

While it is still under debate whether the global area burnt by wildfires increases^16^, modelling studies show that intense fires will occur more frequently in a warming climate^17^. This implies that wildfires will become an increasingly relevant aspect of near-term climate variability in the stratosphere. Currently, however, there are still large uncertainties regarding the climate impact of intense fires^5,18^.

In this study, we investigate the impact of two recent major wildfire events, the Australian wildfires in 2019/20 and the Northern American wildfires in 2017, on the thermal structure of the atmosphere. We present new insights from high-quality satellite observations into changes of the regional vertical atmospheric temperature structure due to the wildfires, as well as the short-term impacts on stratospheric climate.

## Results

**Figure 1:**
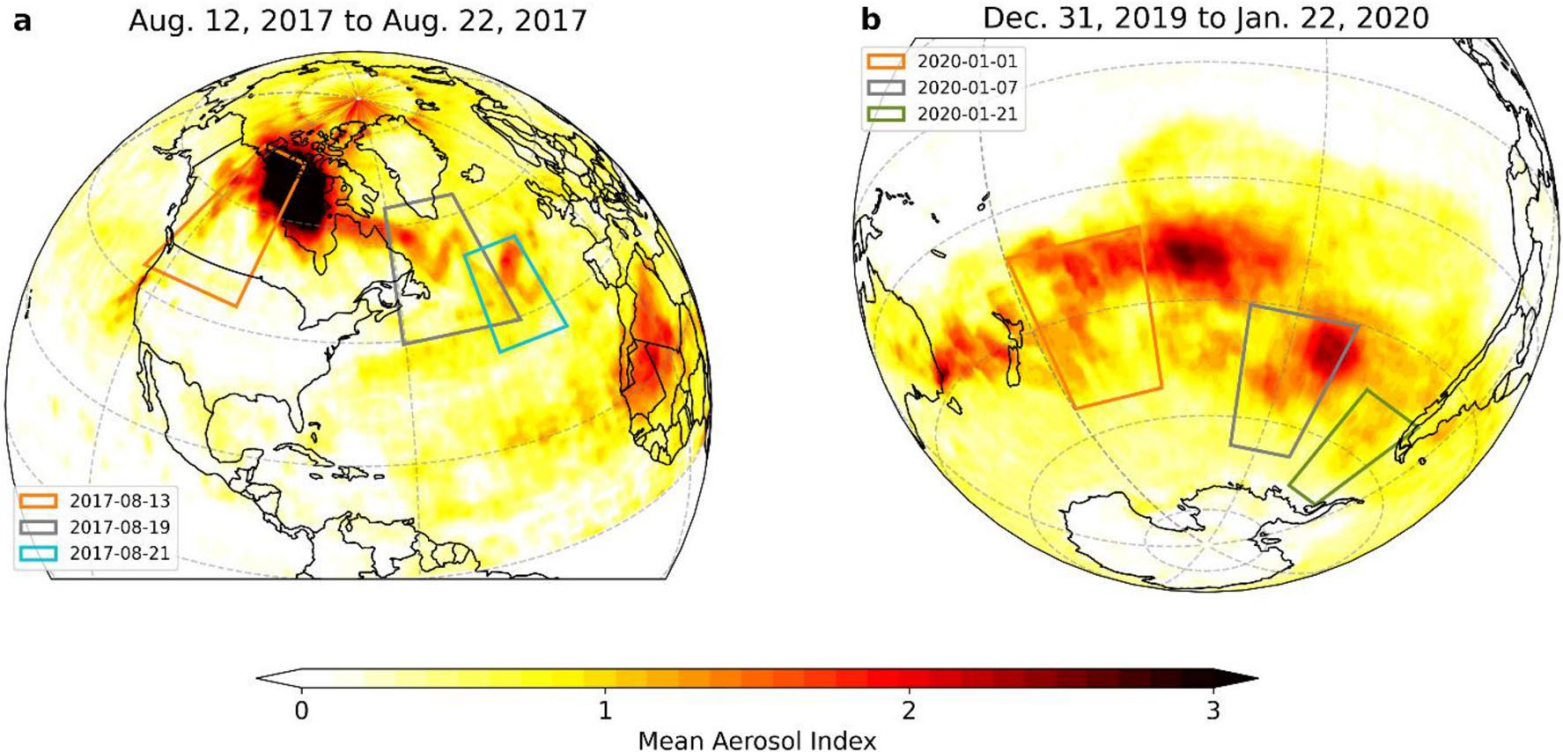
Regions affected by the Northern American and Australian wildfire plumes. (**a**) Mean aerosol index (AI) for the 2017 North American wildfire event between August 12 and 22 and (**b**) the AI between December 31 and January 22 for the 2019/20 Australian wildfire event. Colored rectangles show the area surveyed on the respective days. AI values are plotted for the respective hemisphere where the event occurred. Note that the large AI values measured in the central Pacific did not reach stratospheric heights.

We use global navigation satellite system (GNSS) radio occultation (RO) observations in combination with different aerosol measurements to track the evolution of the wildfire aerosol plumes from day-to-day (Fig. 1) and estimate their short-term climate impact in the upper troposphere and lower stratosphere. Focusing on the impact in the stratosphere, we reveal their immediate effects on the vertical temperature structure by examining RO profiles co-located with the aerosol plume on different days during the first weeks of the wildfires’ development. The regions studied on the different days are indicated in Fig. 1.

### Immediate imprints on the atmospheric temperature structure

The 2017 Northern American wildfire event consisted of multiple pyrocumulonimbus (pyroCb) outbreaks which occurred on August 12 and formed a vast aerosol cloud over Canada at high latitudes during the following days^19^. A portion of this initial aerosol plume was transported eastward across the Atlantic Ocean by the polar jet stream and circled the northern hemisphere within a few weeks^19^. This resulted in a strong perturbation of the aerosol load in the northern hemisphere (cf. Fig. 1a). For estimating the immediate impacts on the temperature structure, we are particularly interested in this part of the initial plume, which reached higher stratospheric altitudes during its early evolution^13,19^.

At the onset of the wildfire, RO temperature profiles associated with the massive aerosol cloud over northern Canada reflect a comparatively warm troposphere and cool tropopause region. Although wildfire aerosols appear to have reached the lowermost stratosphere by this time^13,19^, we do not yet observe a corresponding warming signature. On the subsequent days, a portion of the plume continues to drift eastward and aerosols continue to rise into the stratosphere. However, a majority does not yet exceed the tropopause region.

**Figure 2:**
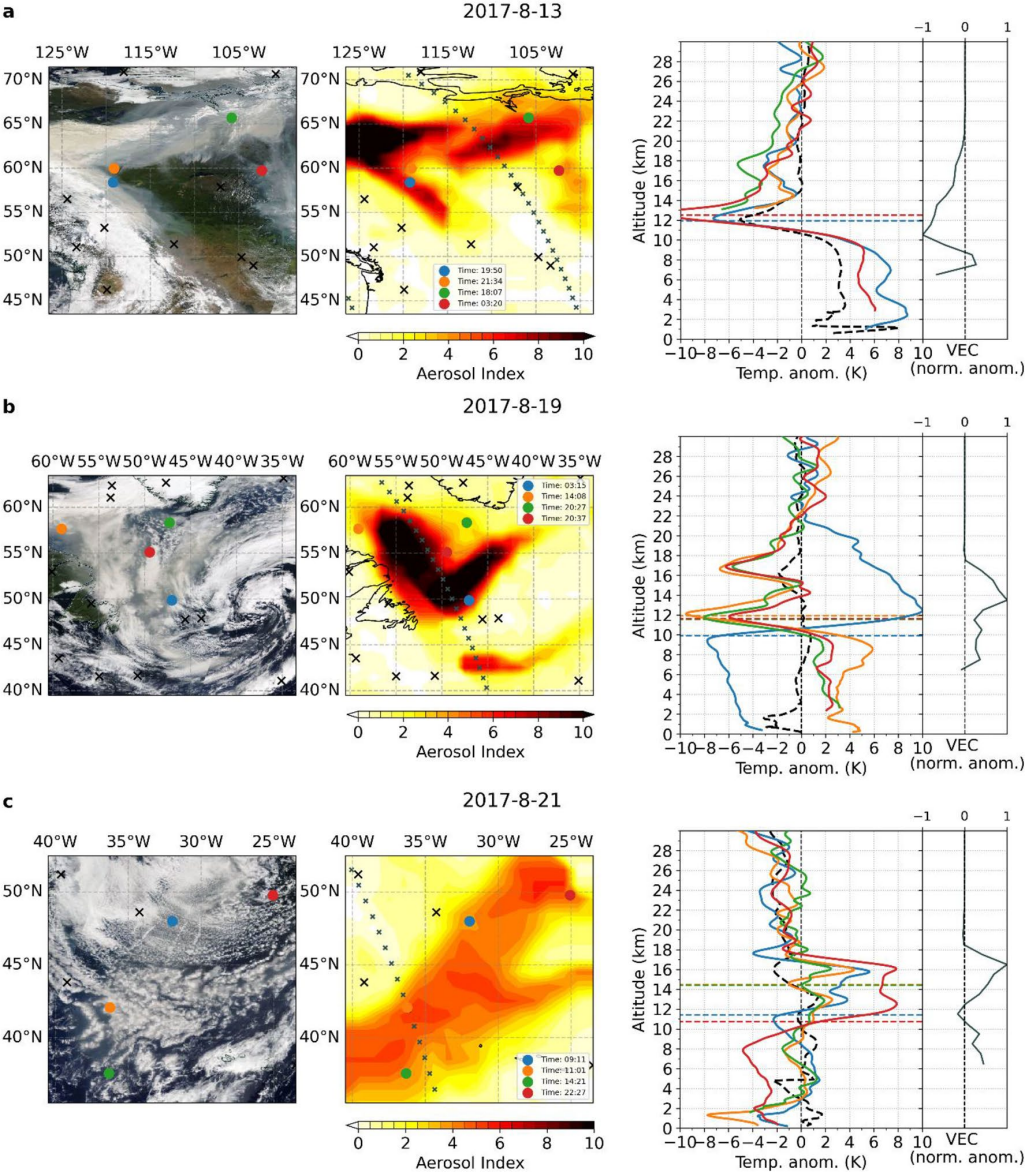
Temperature imprints of the Northern American wildfire plume on different days during its early evolution. MODIS satellite image (left), aerosol index (AI) (center), and RO temperature anomaly profiles (right) located inside the plume (colored lines, right) as well as the mean temperature anomaly profile outside the plume (dashed line) along with OMPS-LP aerosol anomalies (normalized volume extinction coefficient (VEC) relative to the monthly means from 2016 to 2020; right subpanel) for the region investigated on (**a**) August 13, (**b**) August 19 (**b**), and (**c**) August 21 in 2017. Colored dots/black crosses represent the locations of RO profiles inside/outside the aerosol cloud. Small crosses in the central plot indicate the locations of OMPS-LP measurements. Horizontal colored lines in the right plot indicate the lapse rate tropopause for each respective RO profile.

As the plume reaches the Atlantic on August 19, a distinct warming signature becomes evident in the RO profiles recorded within the center of the plume. Warming peaks are observed in the lower stratosphere at an altitude of about 13–16 km (Fig. 2b). Accordingly, aerosol extinction data from the Ozone Mapping and Profiler Suite (OMPS) Limb Profiler (LP) also show a strong maximum at these altitudes (Fig. 2b). This is confirmed by Cloud-Aerosol Lidar and Infrared Pathfinder Satellite Observations (CALIPSO) measurements of the aerosol plume height (Fig. S1b), indicating that the warming is indeed related to wildfire aerosols.

A similar, although weaker, signature is already visible on August 13 for some of the co-located temperature profiles. They show a small warming above the local tropopause (e.g., Fig. 2a, blue profile), which is not observable for profiles in regions less affected by wildfire aerosols (e.g., Fig. 2a, red profile). The aerosol extinction data do not show increased levels above 12 km, probably because the OMPS-LP satellite track does not cross the central plume (Fig. 2a center). However, CALIPSO data show that the plume reached the stratosphere (Fig. S1a) at the time of those RO measurements (blue, orange profile). It is likely that the small lower stratospheric warming signal on August 13 is already related to the wildfire aerosols and could be even stronger within the center of the plume.

The strongest warming during the wildfire plume’s early development, however, is observed as it is further transported across the Atlantic Ocean following August 19. Most RO profiles associated with the plume center reveal a substantial warming of up to 8 K in the lower stratosphere. Depending on latitude, the warming extends up to 16 km and coincides with the OMPS-LP aerosol measurements showing increased aerosol extinction (Fig. 2c).

On its further way, the initially compact aerosol plume becomes less and less dense, and less suitable to be examined using AI data and individual RO profiles.

In the case of the 2019/20 Australian wildfires, the strong pyroCb activity caused a massive increase of the aerosol concentration in the southern hemispheric stratosphere (Fig. 1b), which even exceeded by far the strong stratospheric aerosol optical depth (SAOD) perturbation caused by the 2017 North American wildfires^11,20^.

The first major pyroCb events took place between December 29 and December 31, 2019 and caused a massive aerosol cloud to emerge southeast of the Australian coast (see Fig. 1b). This was followed by a second vast outbreak on January 4^11,21^.

**Figure 3:**
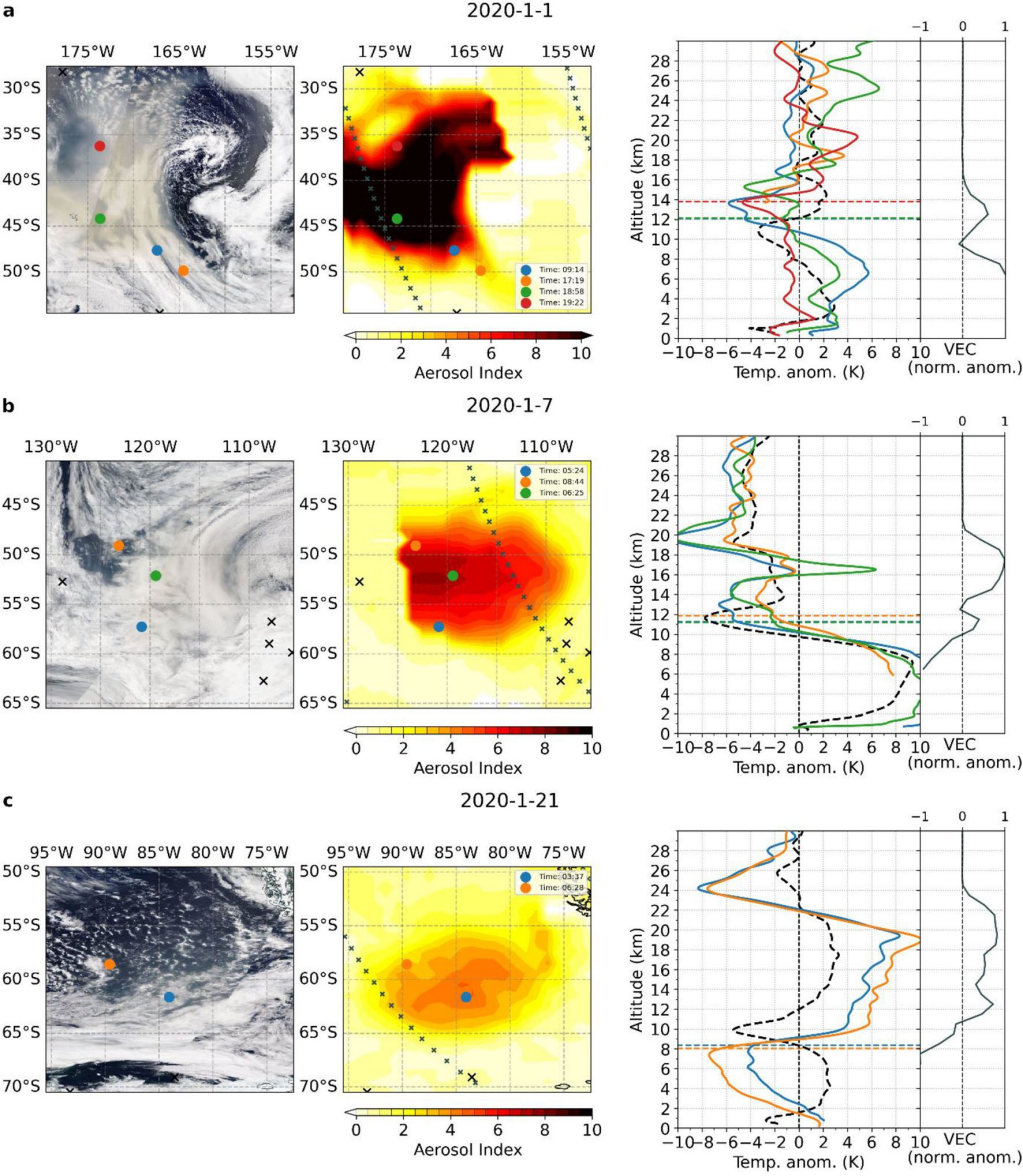
Temperature imprints of the Australian wildfire plume on different days during its early evolution. MODIS satellite image (left), aerosol index (AI) (center), and RO temperature anomaly profiles (right) located inside the plume (colored lines) as well as the mean temperature anomaly profile outside the plume (dashed line) along with OMPS-LP aerosol anomalies (normalized VEC, relative to the monthly means from 2016 to 2020; right subpanel) for the region investigated on (**a**) January 1, (**b**) January 7, and (**c**) January 21 in 2020. Colored dots/black crosses represent the locations of RO profiles inside/outside the aerosol cloud. Small crosses in the central plot indicate the locations of OMPS-LP measurements. Horizontal colored lines in the right plot indicate the lapse rate tropopause for each respective RO profile.

On January 1, the initial plume has already been transported east of New Zealand (Fig. 3a left/middle panel). The co-located OMPS-LP measurements show increased aerosol extinction near an altitude of 13 km (Fig. 3a right subpanel) supported by CALIPSO data (Fig. S2a). Although this height is within or even above the local tropopause region, we find no characteristic warming signal at this early stage of the plume development.

As the aerosol cloud is further transported across the Pacific Ocean the slower-moving part of the plume forms a rapidly rising, dense anticyclonic aerosol vortex^11,20,22^ which can be clearly identified in the AI map (Fig. 3b, middle panel). Now a pronounced warming signature emerges in the lowermost stratosphere in the related temperature anomaly profiles (Fig. 3b, right panel). OMPS-LP aerosol extinction is also strongly elevated at lower stratospheric altitudes. The RO profile recorded within the center of the vortex already shows a distinct positive temperature anomaly in the local stratosphere near 17 km.

Additionally, the RO profiles exhibit a strong negative temperature anomaly above the maximum warming. This cooling does not result from radiative transfer but most likely from a temperature dipole structure related to the thermodynamics of a synoptic scale vortex^11^. For the North American wildfires, a similar vortex structure was found^23^, but the related cooling is less pronounced.

On January 21 (Fig. 3c), as the aerosol vortex from the first pyroCb outbreak reaches the southern tip of South America, the co-located RO profiles show a remarkable warming of up to 10 K. It extends from the local tropopause to the top of the vortex and peaks at an altitude of approximately 20–22 km coinciding with plume heights measured by CALIPSO (Fig. S2c). While on January 7 a stratospheric warming is only detectable within the vortex center, on January 21 also profiles recorded outside the central vortex area display a considerable warming over the whole lower stratospheric altitude range.

For both wildfire events, we constructed continuous timeseries of the temperature profiles co-located with the moving aerosol plumes (Fig. S3), showing the first weeks of the development with the onset of warming in the stratosphere as soon as the aerosols reach the stratosphere.

**Figure 4:**
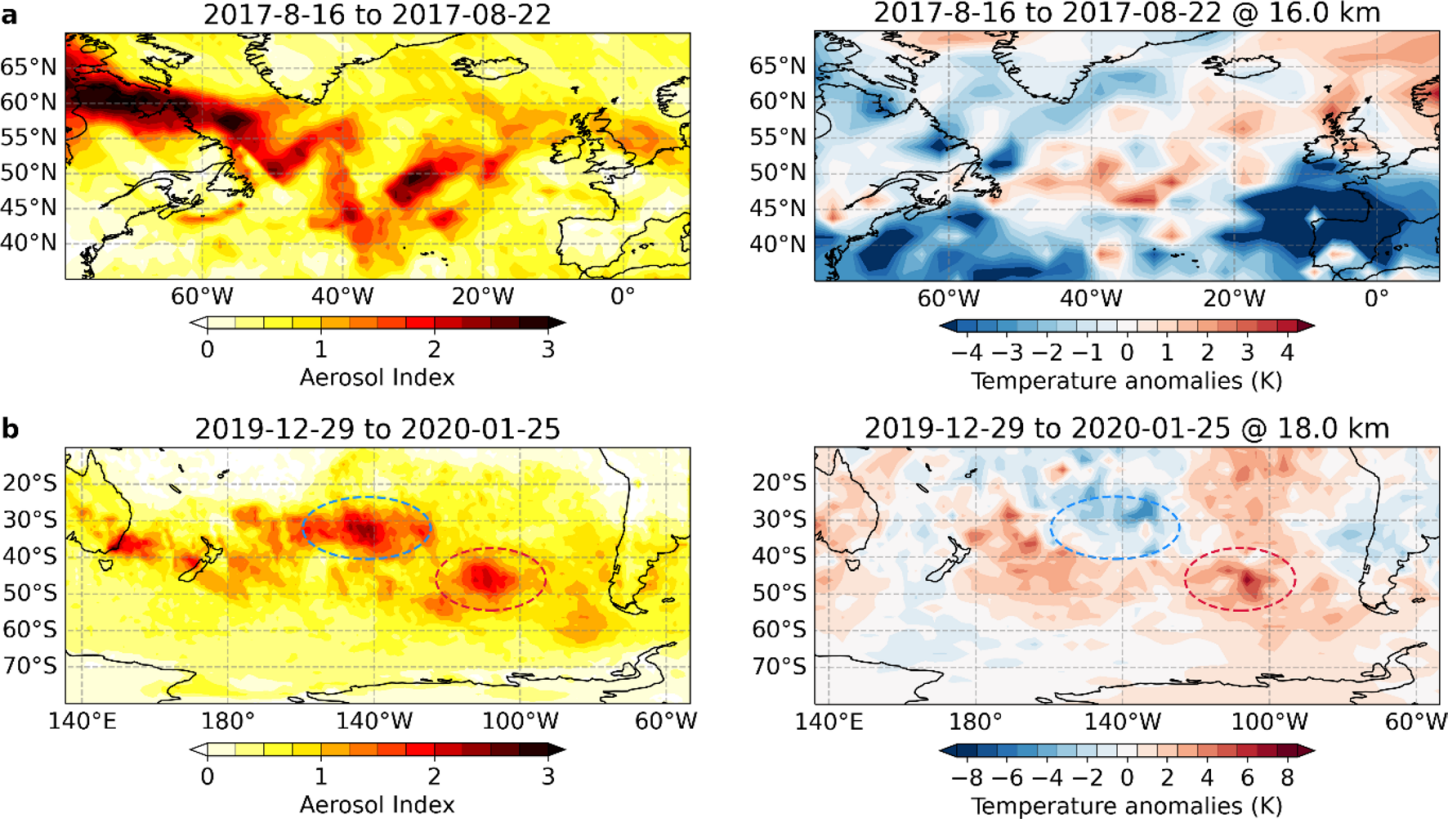
Regional temperature anomalies shortly after the wildfire events. (**a**) Mean aerosol index (AI) and RO temperature anomalies in the lower stratosphere (16 km) in the Northern Atlantic region for the week from August 16 to August 22, 2017. (**b**) Same as (**a**) but for the Southern Pacific region and at an altitude of 18 km for the weeks from December 29, 2019 to January 25, 2020. Note the different temperature ranges in (**a**) and (**b**).

When the aerosol cloud of the North American wildfires crosses the Atlantic in the week from August 16–22, a widespread warming signature in line with the AI pattern is observed in the lower stratosphere (Fig. 4a). The warming is most pronounced at an altitude of 16 km and appears where the strongest wildfire signals are found in the daily RO profiles (Fig. 2bc). The differences in the geographical pattern between AI and RO temperature anomalies can be explained by acknowledging that the mean AI does not contain altitude information of the aerosol pattern, while the temperature anomalies are shown specifically for an altitude of 16 km.

As the AI over Canada mainly represents aerosols which have not penetrated deeper into the lower stratosphere (cf. Fig. 2a), there is no warming imprint visible in stratospheric temperatures over northern Canada. The warming signature over the Atlantic, in contrast, extends all the way from the north-eastern Canadian coast to the British Isles, reaching more than 4 K in some regions.

For the Australian wildfires (Fig. 4b), portions of the aerosol plume have already spread throughout the southern hemisphere during the first four weeks of the event from December 29, 2019 to January 25, 2020. The southern Pacific region, however, is most affected.

A strong heating in the lower stratosphere is observed in most regions affected by wildfire aerosols. The imprint of the aerosol vortex, resulting from the first pyroCb outbreak (Fig. 4b, red ellipse), is also clearly visible and results in a local lower stratospheric warming of up to 8 K. An exception to the overall warming in the southern Pacific region is the negative temperature anomaly at midlatitudes around 18 km altitude (Fig. 4b blue ellipse). This cooling is induced by the second vortex that formed after the second major pyroCb outbreak on January 4. Unlike the first aerosol plume, the vortex center for the second vortex was below 18 km and cooled the local stratosphere above as a consequence of its temperature dipole structure^11,20,22^ shown in Fig. S4.

### Short-term climate imprints

Wildfires not only alter the regional atmospheric temperature structure immediately after the events, but also impact stratospheric short-term climate.

**Figure 5:**
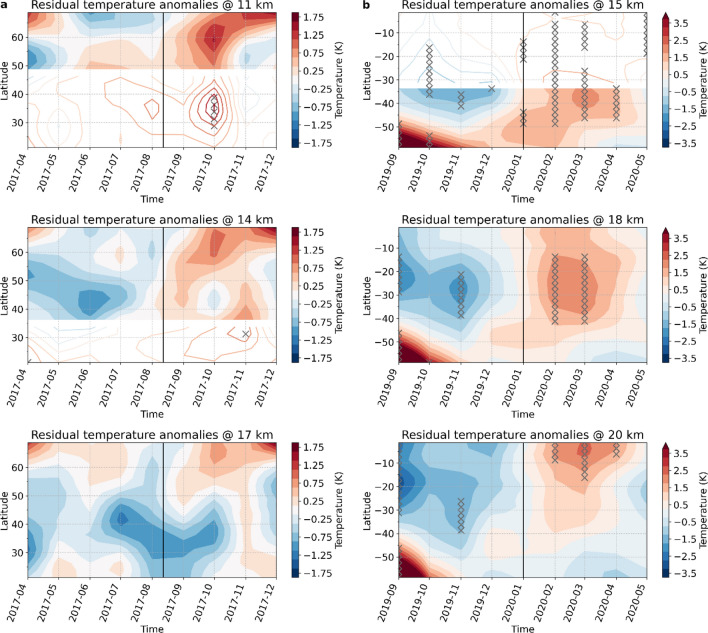
Zonal temperature anomalies before and after the wildfire events. Hovmøller diagram of the residual RO temperature anomalies (QBO and ENSO removed) at three different altitudes for (**a**) the North American and (**b**) the Australian wildfires. Areas that lie below the climatological lapse rate tropopause are shown shaded. The vertical lines indicate the start date of the respective wildfire event. Note the different temperature ranges in (**a**) and (**b**). Values significant at the 95% confidence level are indicated with a cross-mark.

Following the North American wildfires, a persistent warming appears in the zonal temperature anomalies in the lower stratosphere (Fig. 5a). Cool temperature anomalies are prevailing in the stratosphere prior to the wildfire events, and change to warm anomalies shortly after the wildfire occurs. In regions that are less affected by the wildfire aerosol plume, the negative temperature anomaly persists after August 2017.

In mid-latitudes the warming occurs immediately after the event, while at high latitudes the warming signal mainly develops with some delay in September 2017. Positive temperature anomalies which appear to be connected to the Northern American wildfires are observable until October 2017.

For the Australian wildfires, positive anomalies are observable beginning with January 2020 at higher latitudes (60°S to 40°S), peaking at mid to low latitudes by March 2020, and persisting until June (Fig. 5b). Compared to the Northern American wildfires the related temperature anomalies are far more pronounced and statistically significant. During the months before the event, temperature in the lower stratosphere was substantially influenced by a southern hemispheric sudden stratospheric warming (SSW) whose impacts are visible until November 2019. The cooling at mid to low latitudes prior to the event is presumably also a consequence of the SSW^24^.

**Figure 6:**
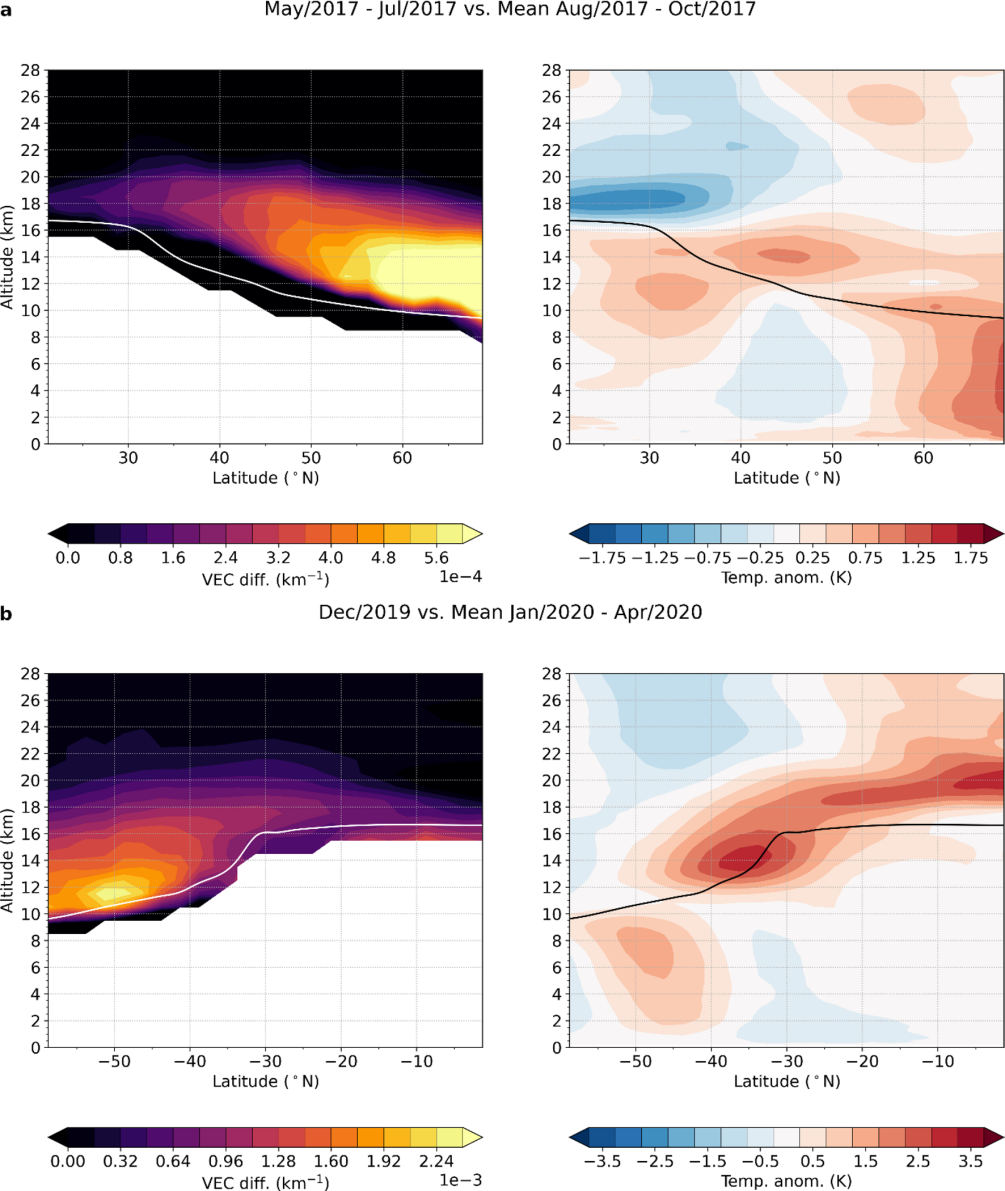
Wildfire induced short-term climate signals. (**a**) Difference in mean aerosol concentration (VEC; left panel) four months before the 2017 North American wildfires vs. three months after the event and the corresponding impact on RO temperature anomalies (right panel). (**b**) Same as (**a**), but for the Australian wildfire in 2019/20. To avoid influence of the southern hemispheric SSW event in 2019, only December 2019 before the event was used for the difference calculation. Note the different ranges for VEC and temperature in (**a**) and (**b**).

In the following, we define the short-term stratospheric climate signal of the North American and Australian wildfires as the difference between the mean temperature anomalies for the months before versus the months after the event (Fig. 6).

For the Northern American wildfires, the aerosol extinction perturbation at mid latitudes is pronounced from mid- to high latitudes (Fig. 6a, left panel). The associated warming signal observed in the RO data, however, is stronger at mid latitudes and reaches up to 1 K at an altitude of 14 km (Fig. 6a right panel). Since it coincides with the warming signature found for this early stage of the event (Figs. 2bc and 4a right panel), it is most likely connected to the elevated part of the aerosol plume, which was initially transported across the Atlantic Ocean. Such a warming behavior that is stronger closer to the equator has already been found for volcanic eruptions^25,26^. However, the absorptive characteristics of volcanic aerosols differ greatly from biomass burning aerosols, which contain substantial amounts of black carbon^15^. Compared to volcanic sulfate which absorbs in the longwave spectrum, black carbon strongly absorbs shortwave solar radiation^14^. Therefore, the higher solar illumination at low to mid latitudes could be an explanation for the strong heating signal in this region. Other components of the wildfire plume could also affect temperature in the stratosphere. Ozone, which has a warming effect, decreased in the lower stratosphere due to the wildfires^10,11,15^, while water vapor, which leads to cooling in the lower stratosphere^27^, is increased^11^. Additionally, simulations show that the diabatic heating predominantly is due to black carbon^15^.

The positive temperature anomaly at high latitudes in the lowermost stratosphere, in contrast, can be related to continuous aerosol entry into the stratosphere from the dense low altitude aerosol plume which persisted over northern Canada (cf. Fig. 1a). This is supported by the fact that in the lower stratosphere at high latitudes the aerosol concentration begins to rise only with September 2017 (Fig. S5).

The short-term climate signal associated with the 2019/20 Australian wildfires (Fig. 6b) reveals a distinct heating of the lower stratosphere at mid to low latitudes, which is strongest around 35°S with about 3.5 K. This is more than three times the warming caused by the 2017 Northern American wildfires. For comparison, this is also substantially larger than the effect of the strongest southern hemispheric volcanic eruption since Pinatubo, the Calbuco in 2015^25^. This impressively reveals the extensive heating potential of wildfire aerosols. Similar to the Northern American wildfires, the maximum heating does not occur where the strongest aerosol perturbation is located but is rather shifted towards lower latitudes with higher insolation during February and March (cf. Fig. S6).

The strong warming in the equatorial region even extends into the northern hemisphere (not shown), which is due to transport of warm air across the equator in response to the strong aerosol heating in the southern hemispheric stratosphere, as shown by model studies^15^^.^

## Discussion

Large wildfires are not only devastating for humans and ecosystems, but can have substantial impact also on Earth’s atmosphere. In our case study of recent strong wildfire events, the Northern American wildfires in 2017 and the Australian wildfires in 2019/20, we observe strong warming signals of up to 10 K in the lower stratosphere as an immediate effect of the aerosol plumes. For the Australian wildfires we find a statistically significant warming signal in the following months, while for the North American wildfires, the short-term climate signal is comparatively weak relative to the natural variability. The short-term impact on climate in the lower stratosphere lasts several months and amounts to about 1 K for the Northern American wildfires, and even up to 3.5 K in southern mid-latitudes for the Australian wildfires.

The results indicate that the imprint from the Australian wildfires is the strongest stratospheric climate signal caused by aerosols since the eruption of the Pinatubo in 1991. While volcanic eruptions occur on an irregular basis and are not influenced by human action, the risk of intense wildfires increases due to climate change^1,17^. Therefore, the impact of wildfires is expected to become increasingly important. Our study clearly reveals a severe impact of large wildfires on stratospheric temperatures, similar as or even larger than volcanic eruptions. This has further implications for stratospheric chemistry, e.g., destruction of stratospheric ozone through the direct impact of aerosols, or through the acceleration of chemical reactions due to a warmer stratosphere.

The temperature signature of wildfires, similar to volcanic eruptions, impacts stratospheric climate variability and affects climate trends. As soon as aerosols reach the stratosphere, they can stay there for years and accumulate, influencing the upper-air climate. This implies that such extreme events will become increasingly important for future climate.

## Methods

### Determining the immediate temperature imprints

During the early stages of the events, we track the wildfire plumes using Moderate-resolution Imaging Spectroradiometer (MODIS) corrected reflectance satellite images in the visible range as well as the UV-aerosol index (UV-AI) from the Ozone Monitoring Instrument (OMI) onboard the Aura satellite.

The UV-AI can be used to detect the presence of UV absorbing aerosols such as soot. It also allows to discriminate between different types of aerosols. Positive UV-AI values indicate the presence of e.g., volcanic aerosols or smoke aerosols while close to zero and negative values indicate clouds or weakly absorbing aerosols such as sea salt^8,28^.

Backscatter measurements from the Cloud-Aerosol Lidar with Orthogonal Polarization (CALIOP) onboard of the Cloud-Aerosol Lidar and Infrared Pathfinder Satellite Observations (CALIPSO) satellite provide profiles of aerosols and clouds with very high vertical resolution^29^^.^

Radio occultation (RO) temperature data which offers high accuracy, very good vertical resolution in the upper troposphere and lower stratosphere (UTLS) region, and global coverage^30–32^, are taken from the Wegener Center (WEGC) occultation processing system version 5.6 (OPSv5.6) multi-satellite record^33^.

Individual temperature profiles as well as monthly-mean temperature data on a 2.5° × 2.5° latitude/longitude grid are utilized. RO profiles which are recorded within a region where the UV-AI exceeds a value of 3 are considered to lie within the center of the aerosol cloud whereas profiles recorded in regions with an UV-AI value lower than 1.5 are considered to lie outside the cloud.

The anomalies of the individual RO profiles are generated by subtracting the values of the nearest grid point in the 2.5° × 2.5° latitude/longitude reference climatology (2002 to 2020) for the corresponding month from the individual profile. A similar method has been successfully applied to detect the vertical thermal structure in tropical cyclones^34^ and after volcanic eruptions^35^. Additionally, weekly 2.5° × 2.5° latitude/longitude temperature anomalies, which are created by subtracting the corresponding climatological month from the weekly data, are utilized.

To check whether the warming signals in the individual RO profiles are related to aerosols, we additionally use vertically resolved Level 2 aerosol data from the Ozone Mapping and Profiler Suite (OMPS). OMPS is a limb profiler (LP) onboard the Suomi National Polar-orbiting Partnership (NPP) satellite which detects limb scattered sunlight. It orbits the earth in a sun-synchronous orbit about 14.5 times a day and takes vertical profiles of the atmospheric limb^36^. The vertical profiles of the aerosol data, which we use to detect the plume top, are calculated as the normalized latitude/longitude means from the OMPS-LP measurements within the investigated region on the individual days.

### Calculation of the short-term climate imprints

For the detection of the short-term climate imprints 2.5°-latitude bands are created from the monthly mean gridded (2.5° × 2.5° latitude/longitude) RO temperature anomalies. The anomalies are created by subtracting the mean seasonal cycle (reference period 2002 to 2020). In addition, variability due to El Niño-Southern Oscillation (ENSO) and Quasi-biennial Oscillation (QBO) is removed by applying multiple linear regression using a generalized least squares with autocorrelated AR(p) error model. QBO is accounted for by using indices derived from the Singapore wind field via empirical orthogonal function (EOF) analysis (the leading four EOFs are utilized)^37^, and ENSO is accounted for by the Niño 3.4 sea surface temperature (SST) index (with a lag of three months). We consider temperature anomalies for individual months to be statistically significant when they lie above two standard deviations of the residual timeseries (95% significance level).

Finally, the short-term climate signal of the event is calculated as the difference between the mean of the residing temperature anomalies for the months before and the months after the event. While a three-month pre-event period is used for the North American wildfires, only December 2019 is used as the pre-event period for the Australian wildfires since the preceding months are affected by the strong southern hemispheric sudden stratospheric warming (SSW) in 2019.

The pre- vs. post-event temperature signals are compared to the pre- vs. post-event aerosol signals. For this purpose, we create gridded monthly aerosol data from the OMPS-LP Level 2 measurements. The aerosol signal is then calculated using the same approach as for the temperature signals.

## Supplementary Information

Supplementary Information.

## Data Availability

The temperature data used in this study are available from the Wegener Center (WEGC) Global Navigation Satellite System (GNSS) radio occultation (RO) record^38^. Vertically resolved Ozone Mapping and Profiler Suite (OMPS) Limb Profiler (LP) Level 2 aerosol extinction data are available from the Atmospheric Research Group at the University of Saskatchewan (https://arg.usask.ca/projects/omps-lp/). UV-aerosol index (AI) data from the Ozon Monitoring Instrument (OMI) were downloaded from the Goddard Earth Sciences Data and Information Services Center (GES DISC) (https://doi.org/10.5067/Aura/OMI/DATA3003). CALIPSO backscatter data and figures (used for Fig. S1 and Fig. S2) are available at the CALIPSO—Data Availability Site (https://www-calipso.larc.nasa.gov/tools/data_avail/). The El Niño-Southern Oscillation (ENSO) and Quasi-biennial Oscillation (QBO) data used in the regression analysis were downloaded from the National Weather Service Climate Prediction Center (CPC) (https://www.cpc.ncep.noaa.gov/data/indices/ersst5.nino.mth.81-10.ascii) and the Freie Universität Berlin (https://www.geo.fu-berlin.de/met/ag/strat/produkte/qbo/), respectively. Processed data used in this study are available from the corresponding author upon request.
