# Supplementary material for: Observing the climate impact of large wildfires on stratospheric temperature
Source: Sci Rep. 2021 Nov 26;11:22994. doi: 10.1038/s41598-021-02335-7 (PMC8626459; doi:10.1038/s41598-021-02335-7)
Supplement: Supplementary file 1 — Supplementary Information. [file 41598_2021_2335_MOESM1_ESM.pdf]

Supplementary Information

Observing the climate impact of large wildfires on stratospheric temperature

Matthias Stocker[1], Florian Ladstädter[1,2] and Andrea K. Steiner[1,2*]

[1]Wegener Center for Climate and Global Change (WEGC), University of Graz, Graz, A-8010, Austria

[2]Institute for Geophysics, Astrophysics, and Meteorology/Institute of Physics, University of Graz, Graz, A-8010, Austria

[*]andi.steiner@uni-graz.at

Supplementary figures

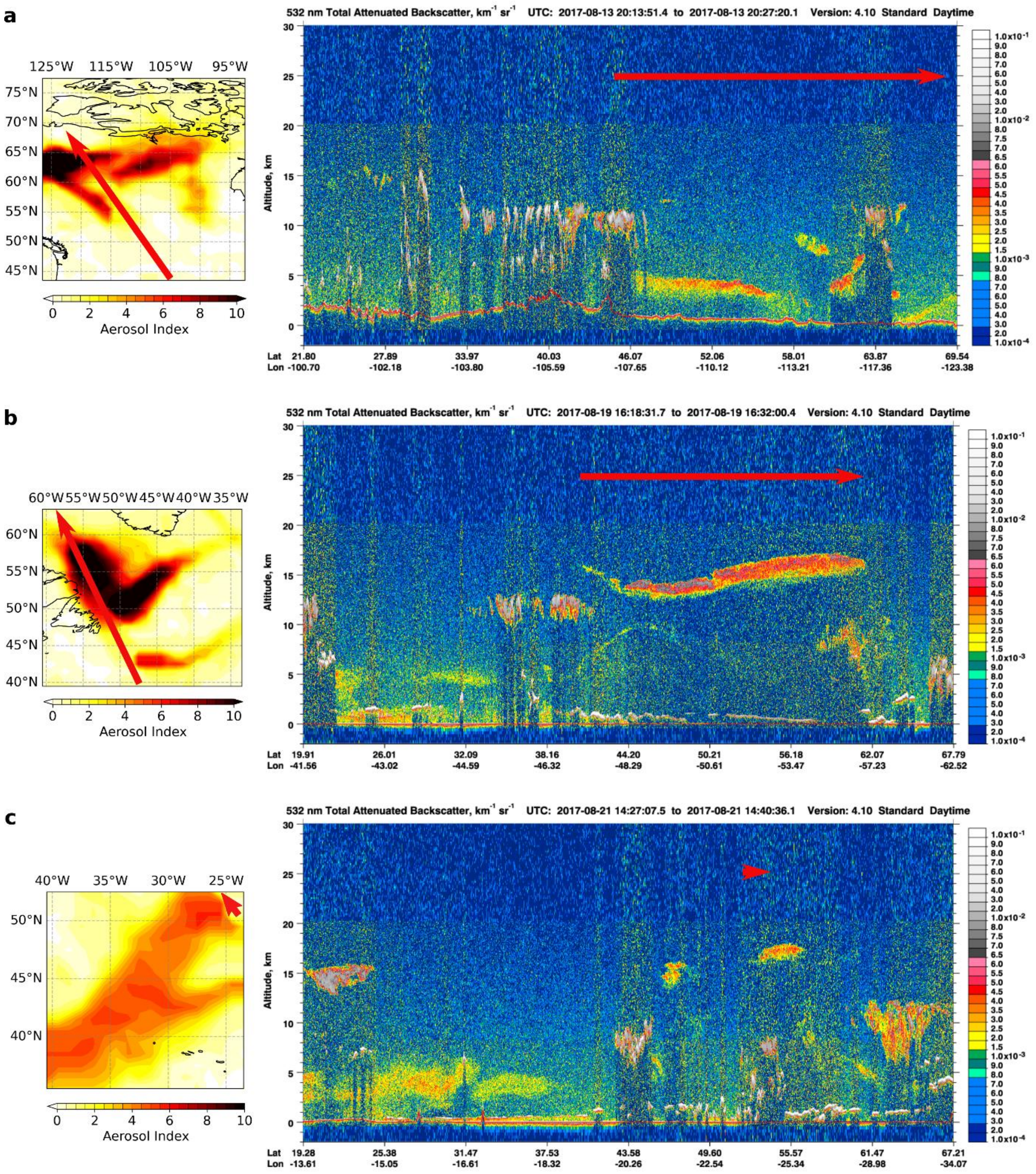

Figure S1: ***CALIPSO backscatter profiles of the Northern American wildfire plume****. Aerosol index (AI, left panels) and attenuated backscatter measurements from CALIPSO co-located with the aerosol plume (right panels) originating from the 2017 Northern American wildfires on (a) August 13, (b) August 19 (b), and (c) August 21 in 2017. Red arrows indicate the region where CALIPSO passed the aerosol plume.*

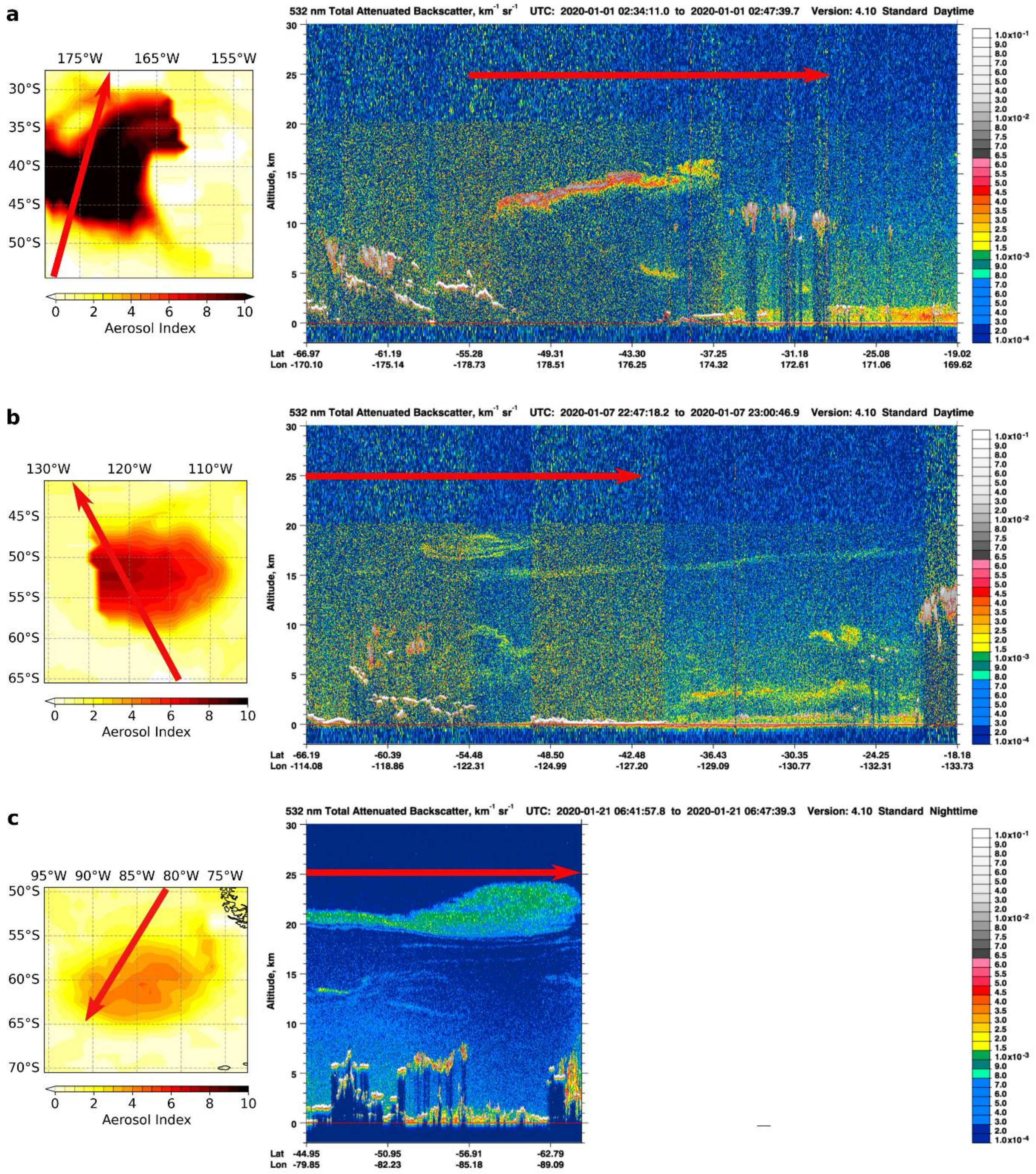

Figure S2: ***CALIPSO backscatter profiles of the Australian wildfire plume.*** *Aerosol index (AI, left panels) and attenuated backscatter measurements from CALIPSO co-located with the aerosol plume (right panels) originating from the 2017 Australian wildfires investigated on (a) January 1, (b) January 7, and (c) January 21 in 2020. Red arrows indicate the region where CALIPSO passed the aerosol plume.*

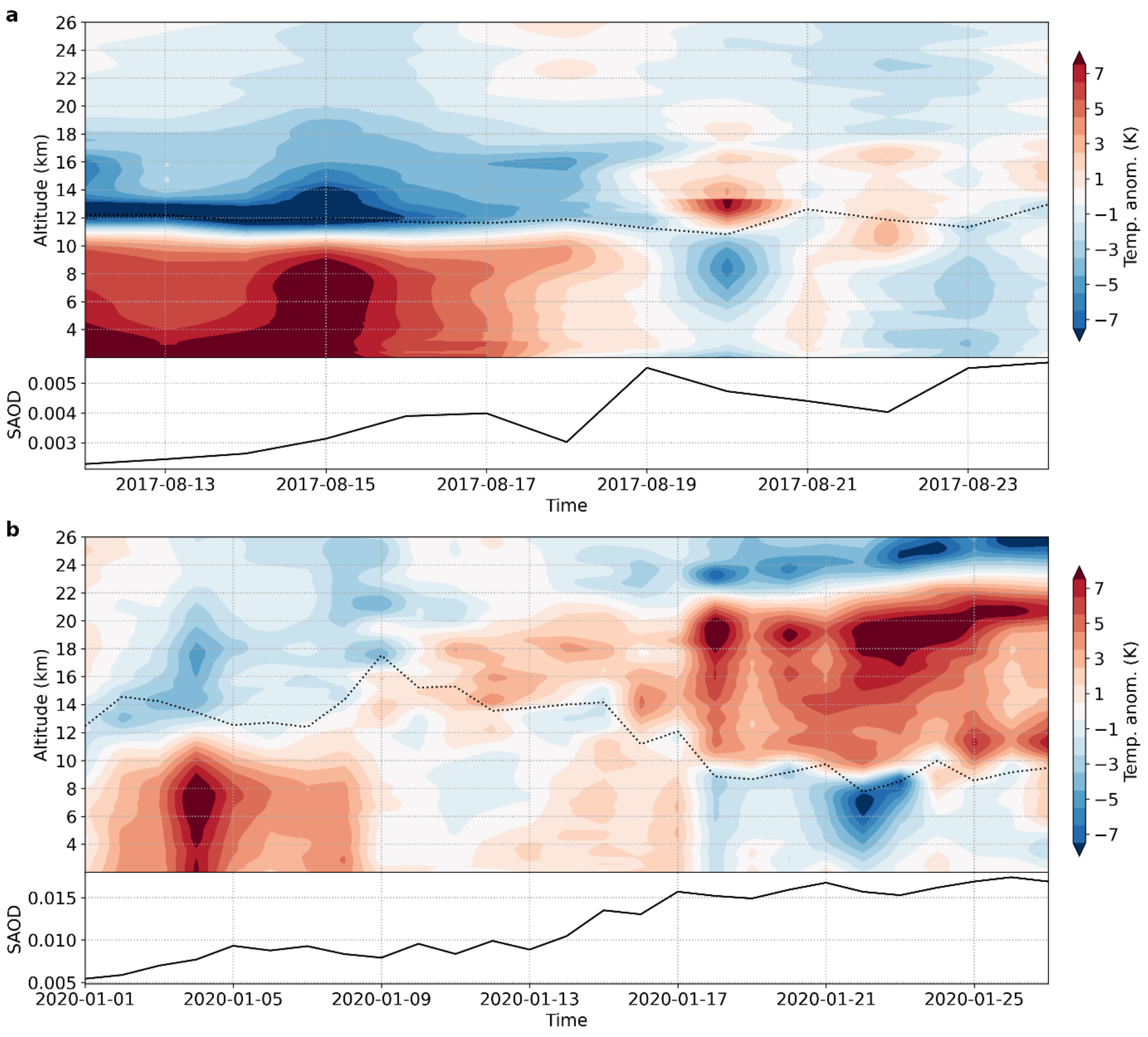

Figure S3: ***Timeseries of the temperature anomalies during the first weeks of the Northern American and Australian wildfires.*** *Mean of the temperature anomaly profiles co-located with the aerosol plumes (to panels) as well as the stratospheric aerosol optical depth (SAOD) (bottom panels) for (a) the Northern American Wildfires in 2017 and (b) the Australian wildfires in 2019/20. The dotted line indicates the mean tropopause altitude computed from the sampled profiles. The SAOD is calculated as the mean value for the region most affected by the wildfire plumes during the first weeks of their development (North Atlantic and Canada; Southern Pacific region).*

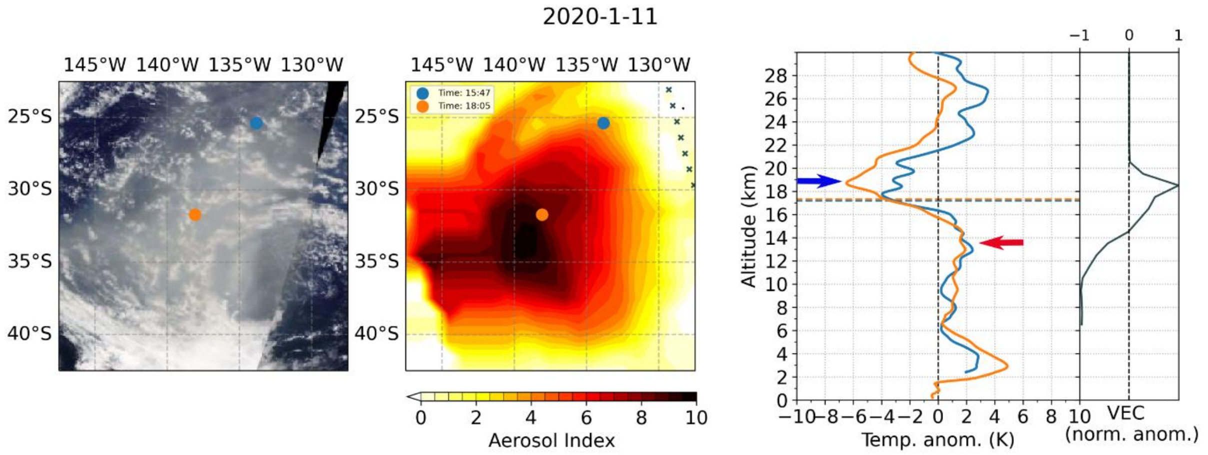

Figure S4: ***Second aerosol vortex following the Australian wildfires.*** *Snapshot of the second aerosol vortex which developed from the second major aerosol plume following the Australian wildfires in 2019/20 as it crossed the central Pacific region at mid-latitudes. MODIS satellite image (left), aerosol index (AI) (center), and RO temperature anomaly profiles inside the plume (right) along with the OMPS-LP aerosol anomalies (normalized VEC; right subpanel). The blue arrow indicates the negative temperature anomaly in the lowermost stratosphere and the red arrow indicates the positive temperature anomaly in the upper troposphere, due to the second aerosol vortex occurring below 18 km altitude.*

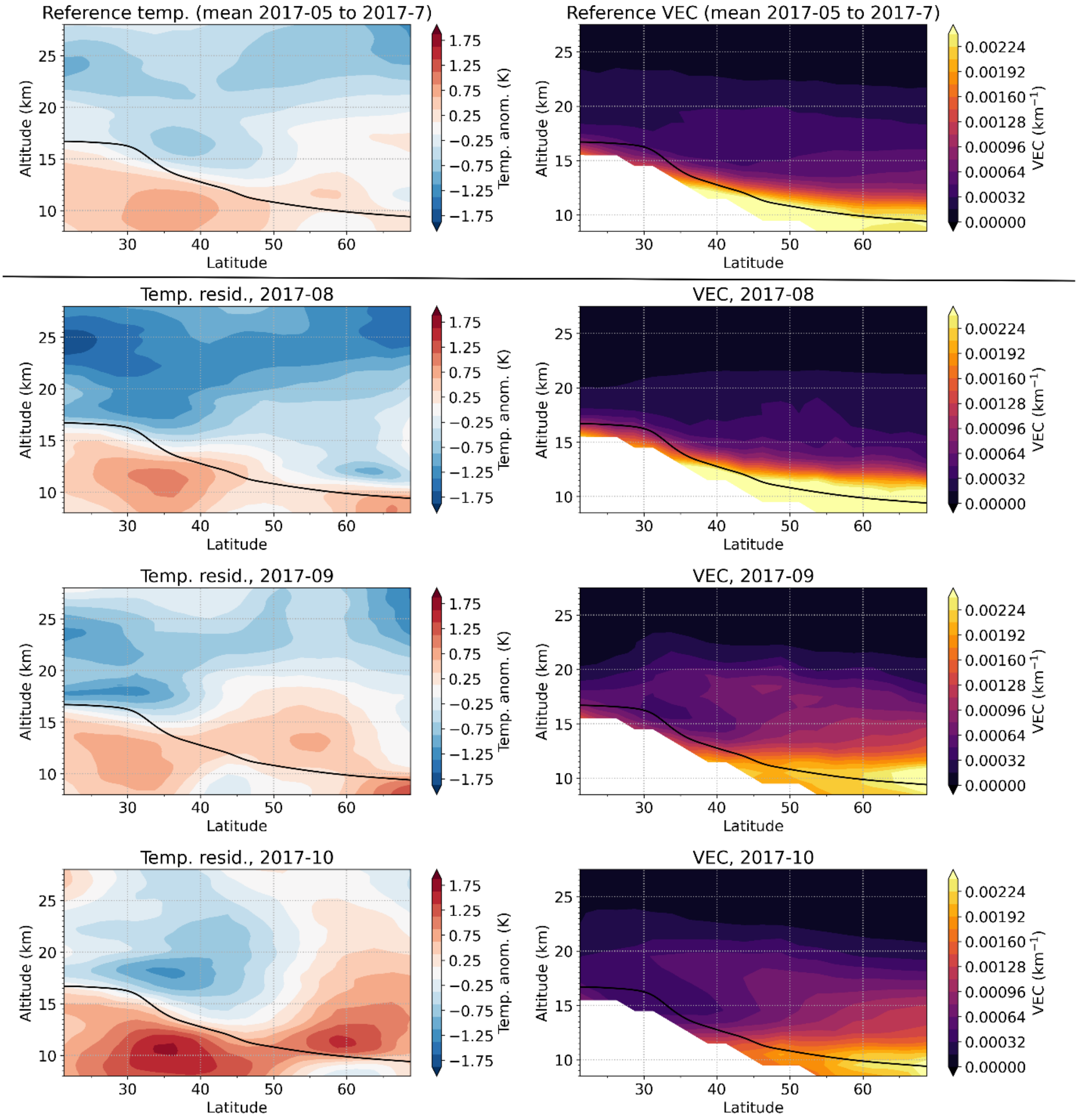

Figure S5: ***Individual months after the North American wildfires compared to the reference period before****. RO temperature anomalies (left panels) and aerosol extinction (VEC; right panels) for the months following the Northern American wildfires in 2017. The top panels show the reference temperature anomalies and aerosol extinction, which are the mean for three months prior to the event.*

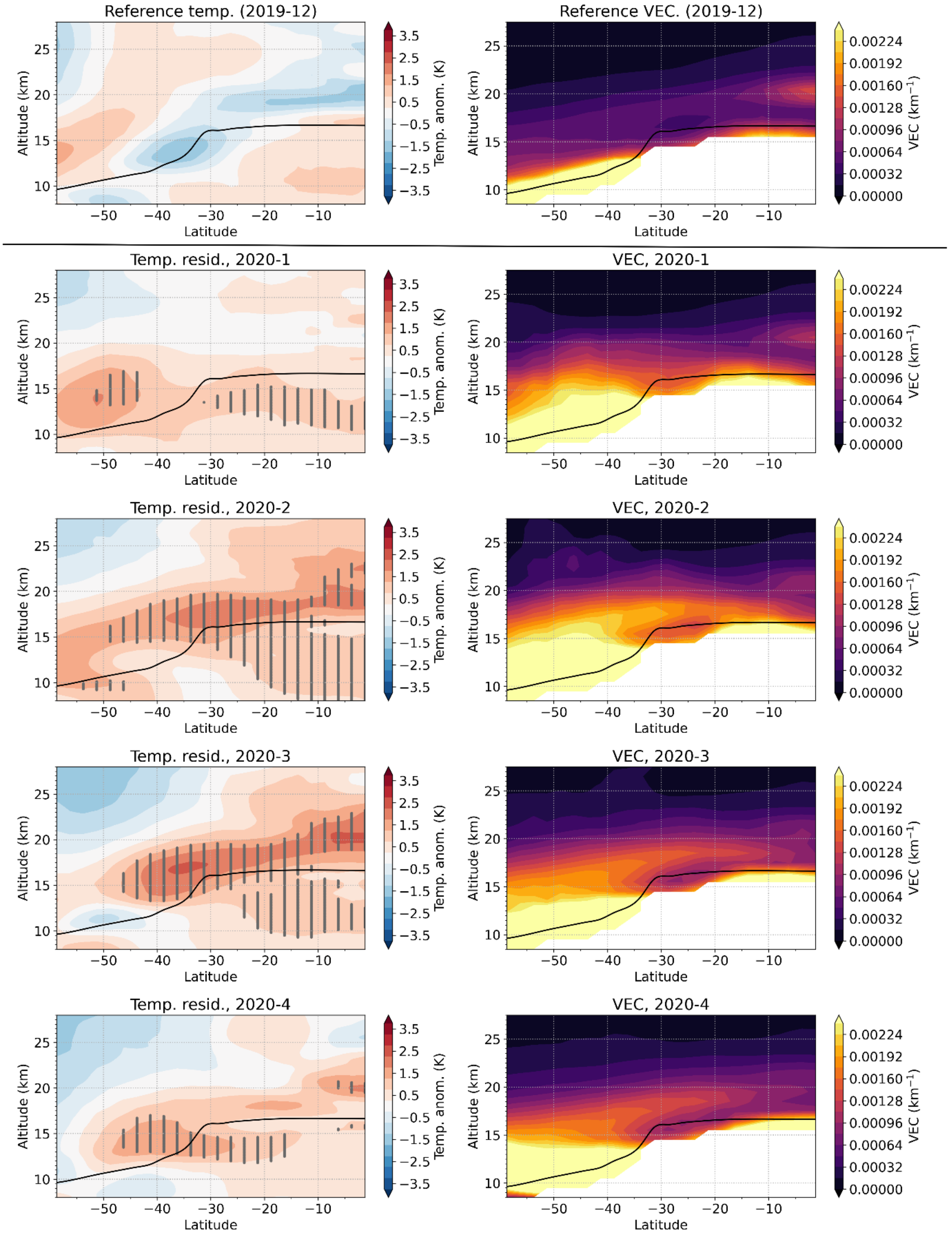

Figure S6: ***Individual months after the Australian wildfires compared to the reference period before.*** *RO temperature anomalies (left panels) and aerosol extinction (VEC; right panels) for the months following the Australian wildfires in 2019/20. The top panels show the reference temperature anomalies and aerosol extinction prior to the event (Dec. 2019). Values that are significant at the 95% confidence level are marked.*
